# Predicted Impact of Climate Change on Trihalomethanes Formation in Drinking Water Treatment

**DOI:** 10.1038/s41598-019-46238-0

**Published:** 2019-07-10

**Authors:** Maria Valdivia-Garcia, Paul Weir, David W. Graham, David Werner

**Affiliations:** 1 School of Engineering Newcastle University, Newcastle upon Tyne, United Kingdom; 2 Scottish Water, Castle House, Dunfermline, Edinburgh, United Kingdom

**Keywords:** Civil engineering, Risk factors, Environmental health

## Abstract

Quantitative predictions of impacts on public water supplies are essential for planning climate change adaptations. Monitoring data from five full-scale Scottish drinking water treatment plants (DWTPs) showed that significant correlations exist between conditionally carcinogenic trihalomethanes (THMs) levels, water temperature (r = 0.812, p = 0.0013) and dissolved organic carbon (DOC) (r = 0.892, p < 0.0001), respectively. The strong seasonality of these parameters demonstrated how climate can influence THMs formation. We quantified with laboratory experiments the sensitivity of THMs formation to changes in water temperature and DOC concentration. The laboratory data accurately reproduced real-world THM formation in the DWTPs. We then combined these validated relationships with information from the literature about future trends in mean summer temperatures and surface water DOC in the British Isles, to estimate future global warming impacts on THMs formation in DWTPs that use chlorine for disinfection. An increase in mean summer temperatures will likely increase THM formation, with a 1.8 °C temperature increase and 39% THMs increase by 2050 representing our mid-range scenario. Such an increase has major implications to potable water around the world, either an increased health risk or increased water treatment costs to maintain an equivalent quality potable supply.

## Introduction

Climatic conditions are changing^1^, and this appears to be altering surface water quality with potential public health implications, especially water treatability in drinking water treatment plants (DWTPs)^2,3^. This becomes particularly evident in regions with extensive peatlands in drinking water catchments where warming effects have been related to increasing dissolved organic carbon (DOC) concentrations, which then impacts water treatability in DWTPs^4–8^. DOC is a precursor for the formation of disinfection by-products (DBPs), some of which are regulated due to public health concerns. For example, conditionally carcinogenic trihalomethanes (THMs) are formed as by-products when water is chlorinated to kill pathogens^9,10^. The European Union regulates total THMs in drinking water (comprising chloroform, bromodichloromethane, dibromochloromethane and bromoform) at 100 μg/L^11^, whereas the US EPA imposes a maximum annual average of 80 μg/L for these compounds^12^.

According to Evans *et al*.^6^ DOC concentrations in 22 UK upland waters have increased by an average of 91% over 15 years. Similar concurrent DOC increases have been recorded elsewhere in Europe and North America^13^. In a detailed analysis of various potential causes of raising DOC in UK lake and streams, Evans *et al*.^14^ identified declining anthropogenic sulphur emission and deposition as the main cause. DOC solubility is suppressed by high soil water acidity, which has lessened in the UK as a result of declining sulphur emission and deposition since the 1980s^15^. However, rising DOC trends were also observed at sites, where non-marine sulphate changes were small, such as Loch Coire nan Arr in Northwest Scotland. Overall, Evans *et al*.^14^ concluded that increases in temperature are the second most relevant cause, and could account for a 10–20% DOC increase.

The dramatic increase in DOC as a THM formation precursor in UK surface waters has implications for drinking water providers, which are challenged to adapt their treatment trains and operations to maintain compliance with drinking water standards into the future^16,17^. In this context, water providers need to anticipate the likely magnitude of further climate change effects in order to effectively plan their investment strategy and adaptations^18^.

Since water providers regularly adapt and optimize their treatments for economic reasons and in response to regulatory changes and technological developments, it is difficult to relate directly long-term trends in surface water composition and ambient temperature to those observed for THMs in potable water. In this study, we took a different approach by considering seasonal trends in surface water quality and potable water THMs over a calendar year for five DWTPs works, which did not undergo major treatment changes or upgrades during this observation period. It has been previously noted that strong seasonal trends in THMs, with maxima in late summer and minima in early spring, and positive correlations between ambient temperature, raw water DOC, and THMs indicate the susceptibility of drinking water quality to changes in climatic conditions^17^. Furthermore, monitoring and chlorination experiments have established that temperature and DOC are influential in THM formation^19,20^. However, to the best of our knowledge no study has yet attempted to reproduce seasonal trends in THMs in treatment works based on independent laboratory experiments.

We used correlation analysis and extensive monthly monitoring data from five full-scale DWTPs in Scotland to first firmly establish changing DOC and ambient temperature as the main causes for seasonality in THMs formation. We next used controlled laboratory experiments to investigate, for each correlation factor separately, how variations in water temperature and DOC concentration will influence THM formation. We then derived from the laboratory data a quantitative relationship between the changes in water temperature, water DOC and THMs formation, and validated this relationship with the monitoring data from the DWTPs. By combining the insights thus gained with climate change scenarios published in the literature, we were finally in a position to discuss the likely magnitude of future climate change impacts on THMs formation in DWTPs. While this work is particularly relevant for drinking water treatment in regions with extensive peatlands in drinking water catchments, the broader implications apply globally to DWTPs using chlorine for disinfection.

## Results

### Correlation analysis of seasonal changes in water quality and THMs at five case study sites

**Figure 1:**
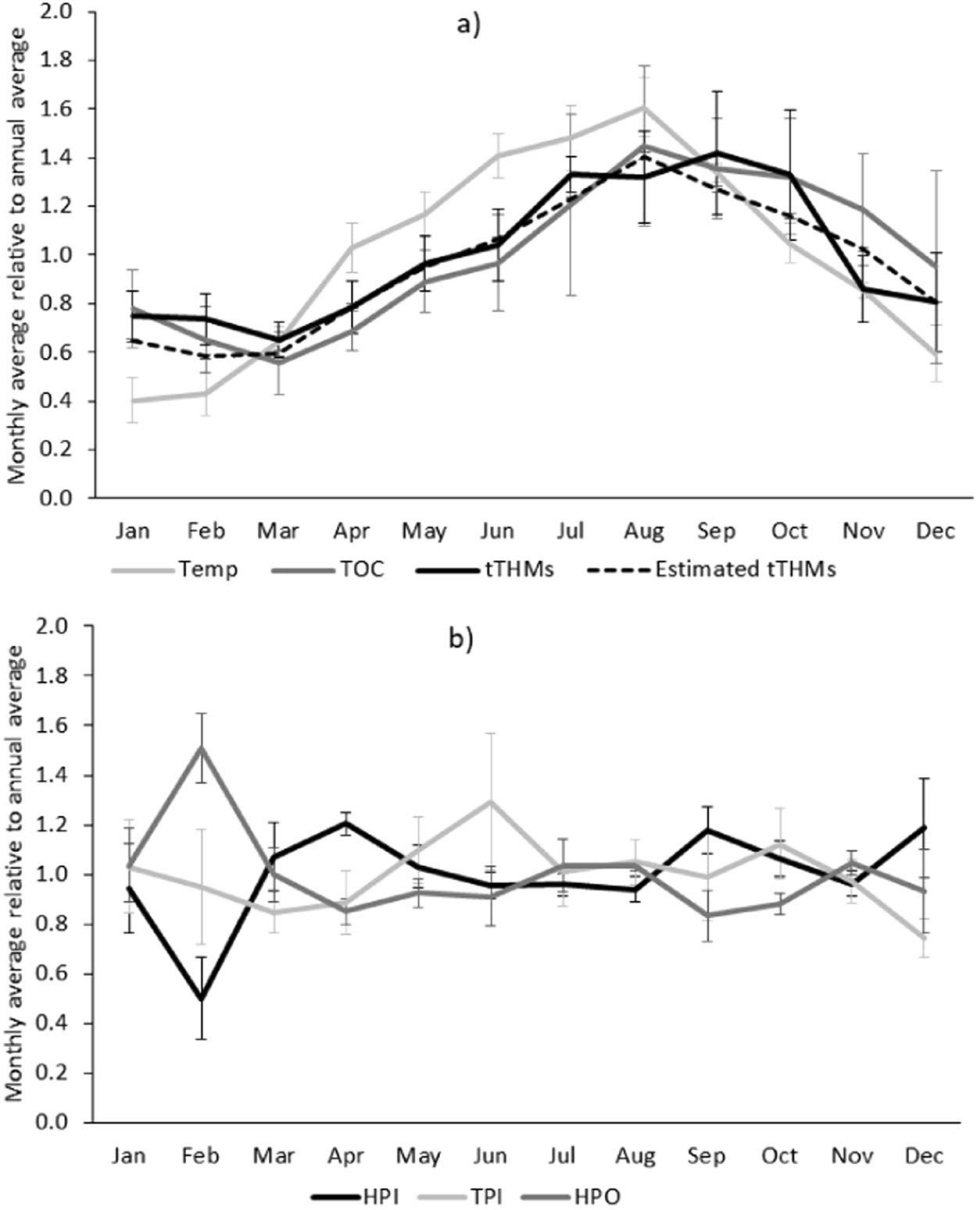
Seasonality in (**a**) the average potable water quality, and (**b**) the average dissolved organic carbon (DOC) composition, expressed as % hydrophobic organic fraction (HPO), transphilic organic fraction (TPI) and hydrophilic organic fraction (HPO), in potable water of five Scottish water treatment plants. Error bars indicate the standard error (n = 5) for the monthly average, relative to the annual average, calculated for the case study sites. Annual average values were 9.66 °C for temperature, 1.08 mg/L for TOC, 43.93 mg/L for THMs, 40.1% for HPI, 18.6% for TPI and 41.3% for HPO.

THMs in potable water from the five case study Scottish DWTPs described in Table S1 in Supplementary Information showed strong seasonality (Fig. 1a). The peak THM concentrations measured in September were on average more than twice as high as the minima measured in March. Our monthly monitoring of water quality at the five case study sites in Scotland comprised 42 parameters of potable water quality and 38 parameters of raw water quality. However, amongst the many parameters monitored (summarized in Table S2 & S3 in Supporting Information), only the monthly averages of water temperature, and various indicators of the water organic carbon content (TOC, HPI and TPI, transmission at UV wavelengths 254 and 270 nm) had statistically significant Pearson correlations (p < 0.05) with the monthly averages of total THMs (Table 1). The significant correlation between water temperature and TOC (0.689, p < 0.05) motivated us to investigate the effect of each factor on THMs separately, as described below, to assure their relationships with THMs were not spurious. Observed correlations were generally strongest for chloroform, and then became weaker and/or statistically insignificant with increased bromination of the THMs. Average bromide concentrations in raw waters at case study sites varied from 19 to 173 mg/L, and from 8 to 81 mg/L in potable water. The THM speciation varied accordingly, with brominated THMs being more prevalent at higher bromide concentration, as would be expected^21^. Further, there were very strong and significant positive correlations between potable and raw water temperature (r = 0.989, p < 0.0001). Equally, there were statistically significant strong and positive correlations between potable and raw water TOC (r = 0.845, p = 0.0005 for unfiltered raw water TOC, r = 0.938, p < 0.0001 for filtered raw water TOC, equivalent to DOC), showing similar seasonality for these parameters in raw and treated water. This is despite substantial TOC reductions during water treatment, by an average 87.0 ± 0.7%. We also analysed seasonal changes in indicators of organic carbon quality in potable water, such as the percentage of organic matter belonging to the hydrophobic organic fraction (HPO), the transphilic organic fraction (TPI), and the hydrophilic organic fraction (HPI). However, no clear seasonal trends in these indicators of organic carbon quality were apparent in potable water (Fig. 1b) that would correspond to the seasonality observed for the THMs (Fig. 1a), and there were no statistically significant Pearson correlations between monthly averages of the percentage of TOC belonging to each of the HPO/TPI/HPI fraction and total THMs.

**Table 1:** Statistically significant linear Pearson correlations between monthly averages of the total or individual THMs and other water quality parameters in potable water from five Scottish water treatment plants (n.s. = p > 0.05, CHBr_2_Cl and CHBr_3_ had no significant correlations).

Parameter	tTHMs	CHCl_3_	CHBrCl_2_
Temp (°C)	0.812 (p = 0.0013)	0.726 (p = 0.0075)	0.833 (p = 0.0008)
TOC (mg/L)	0.892 (p < 0.0001)	0.943 (p < 0.0001)	0.690 (p < 0.0130)
HPI (mg/L)	0.731 (p = 0.0070)	0.804 (p = 0.0016)	n.s.
TPI (mg/L)	0.876 (p = 0.0002)	0.953 (p < 0.0001)	0.661 (p < 0.0192)
T254 nm	−0.773 (p = 0.0032)	−0.870 (p = 0.0002)	n.s.
T270 nm	−0.696 (p = 0.0120)	−0.823 (p = 0.0010)	n.s.

### THMs formation in laboratory experiments

In the months of March, June, September and December water samples were obtained from the five DWTPs to separately investigate, with controlled laboratory experiments, the effects of organic carbon concentration and temperature, and hypothetical changes in DOC quality between the months on THMs formation. The raw water from the case study sites was diluted to either 5 or 1 mg/L DOC concentrations (to create common chemical conditions for parallel experiments) before disinfection with a fixed amount of chlorine at a fixed temperature of either 5, 15, or 25 °C. The treated water from the case study sites sampled before the point of disinfection was diluted to 1 mg/L DOC concentration, if it exceeded this concentration level. By repeating these experiments in the months of March, June, September and December, the potential influence of seasonal changes in DOC composition on THM formation could be investigated.

**Figure 2:**
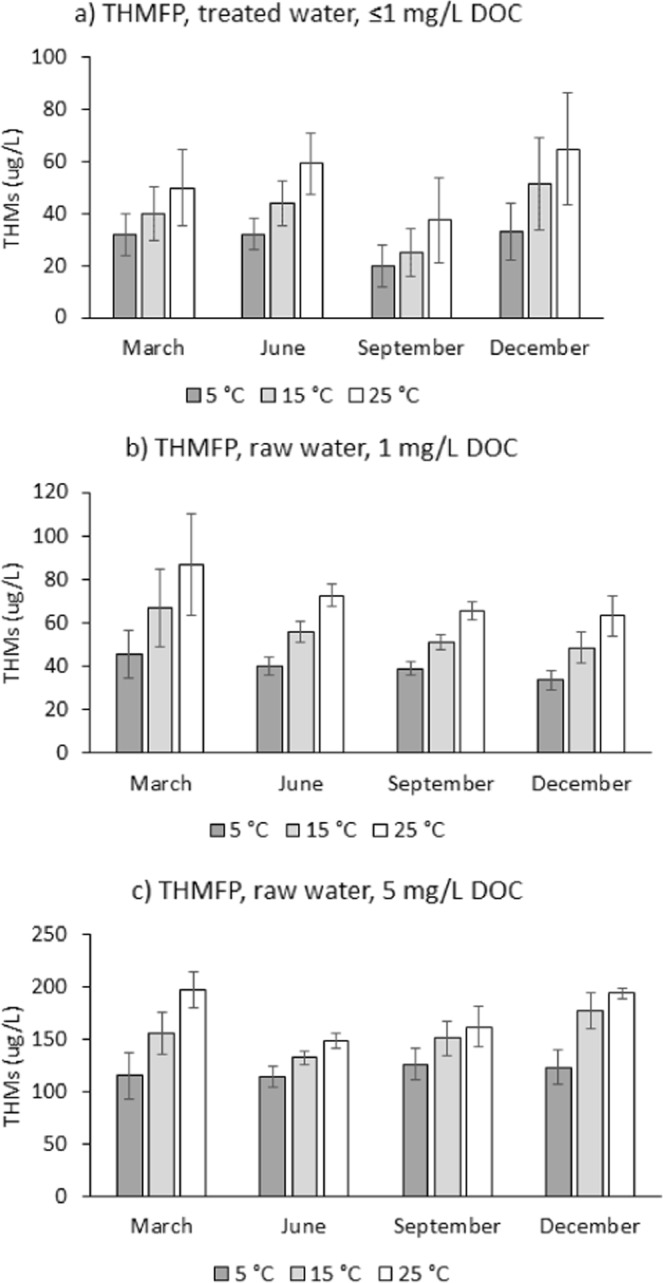
Average trihalomethanes formation potential (THMFP) in (**a**) treated (potable) water sampled from five Scottish water treatment plants before the point of disinfection, and their raw water, diluted to a dissolved organic carbon (DOC) content of (**b**) 1 mg/L and (**c**) 5 mg/L. Error bars indicate the standard errors (n = 5) for the average THMFP of water from the case study sites described in Table S1 in Supporting Information.

**Figure 3:**
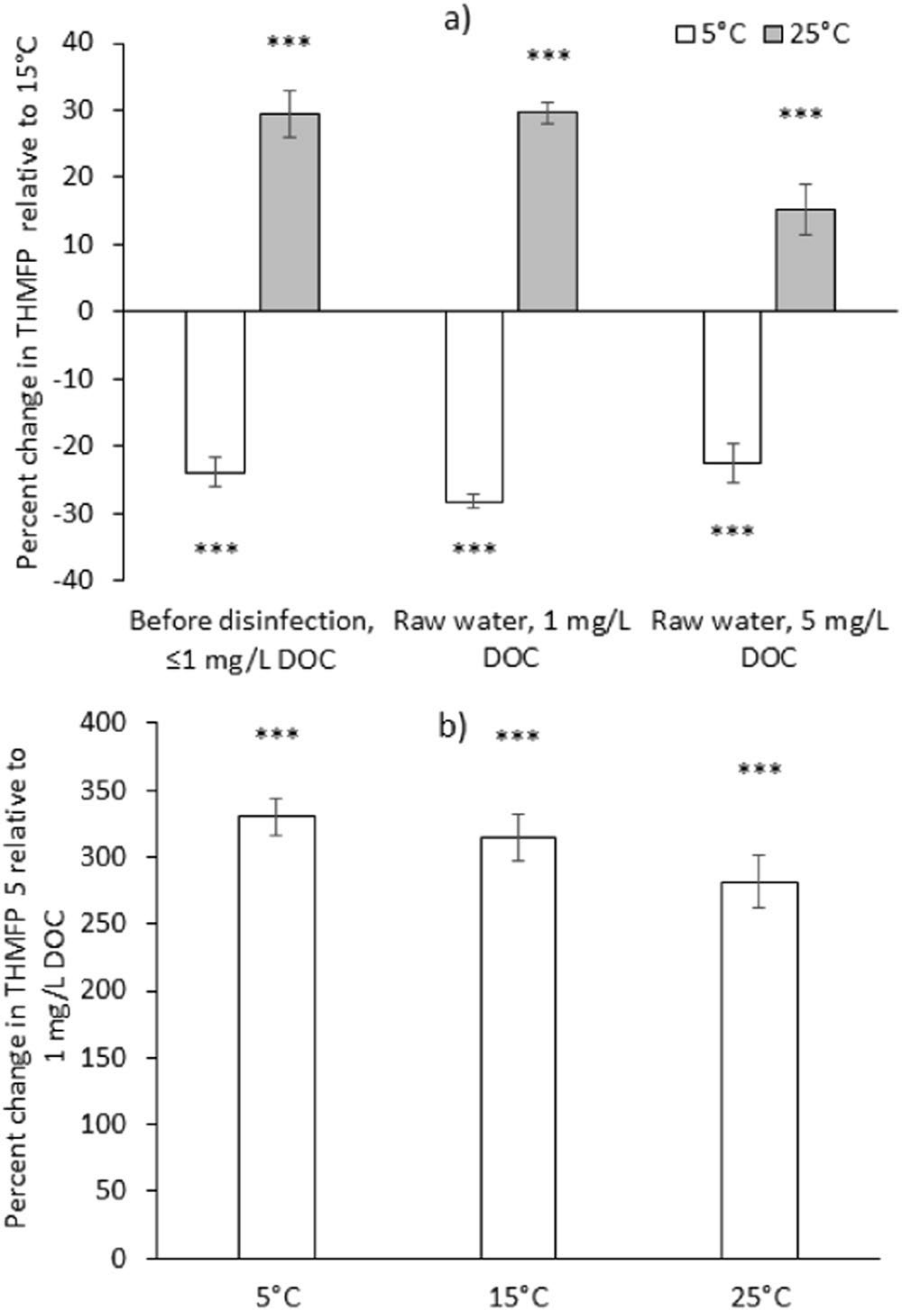
(**a**) Temperature and (**b**) DOC concentration effect on the trihalomethanes formation potential (THMFP) in laboratory experiments for water sampled from the five case study sites. Error bars indicate the standard errors (n = 20) between the measurements for waters from different sites and seasons, while stars indicate the significance of the z-test results for the observed change being different from zero (***p ≤ 0.001).

From the laboratory test results (Fig. 2) it was evident that water temperature directly influences THMs formation, as more THMs were consistently formed for a fixed amount of DOC of the same origin, at higher water temperature. This is in line with expectations for how temperature affects the reaction kinetics leading to THM formation^20^. On average, 24 ± 2% less THMs were being formed in potable water at 5 °C (a typical water temperature in the winter months in Scotland), as compared to 15 °C (a typical water temperature in the summer months in Scotland), whereas 29 ± 4% more THMs were being formed at 25 °C as compared to 15 °C (Fig. 3a), all statistically significant differences (z-test versus 0% difference, two tailed, n = 20, all p < 0.0001). Similarly, our previously published multi-linear regression model derived from THM measurements in 93 Scottish WTPs suggests that a 10 °C increase in water temperature from the annual average of 8.8 °C would increase annual average THM concentrations by 27%^17^. THMFP experiments with raw water diluted to 1 mg/L and 5 mg/L DOC yielded similar results. The magnitude of the temperature effect on THM formation was generally reduced with increased bromination of the THM compounds (Fig. S1 in Supplementary Information). This could be because, in addition to temperature, bromide availability may also control the reaction kinetics for the formation of brominated THM compounds^9^.

As expected, a lower content of the THM precursor organic carbon in water led to less THM formation during disinfection with chlorine (Fig. 2b versus 2c). For 5 mg/L instead of 1 mg/L raw water DOC, THM formation at a fixed temperature of 15 °C was significantly higher by an average 315 ± 18% (Fig. 3b). The increase in THM formation was less than the 500% increase in DOC concentration, which may be due to other limiting factors involved in the complex THM formation reaction kinetics^9^, such as bromide. Indeed, the measured increase in the formation of individual THM compounds at 15 °C was 575 ± 75% for chloroform, but only 49 ± 14% for bromoform (Fig. S2 in Supporting Information). In our experiments we kept bromide concentrations constant by using a salt buffer solution to dilute DOC. Similarly, our previously published multi-linear regression model derived from THM measurements in 93 Scottish WTPs^17^ suggests that a 500% increase in water DOC from the annual average of 1.7 mg/L would increase annual average THM concentrations by 352%. The variability of the THMFP measured for a fixed amount of 1 mg/L DOC across the different case study sites indicates that DOC quality is to some extent influential in THM formation (Tables S4–6 in Supplementary Information), since it is the only obvious variable between these treatments. However, there is no evidence for changes in DOC quality driving THM seasonality. There was no evidence for higher THM formation per mg DOC in the month of September, when seasonal THM formation peaks, as compared to the other months (Dec, March, June, Fig. 2), and the DOC quality indicators HPI, TPI and HPO were not correlated with total THMs in the monitoring data from the case study sites, if expressed as % of DOC (Fig. 1b), even though absolute HPI and TPI concentrations were positively correlated with total THMs (Table 1). Golea *et al*.^22^ also concluded that changes in disinfection by-product levels from the chlorination of Scottish waters can mainly be attributed to the concentration of reactive natural organic matter (NOM), rather than changes in NOM reactivity.

### Predicted versus measured seasonality in THMs

The laboratory data was used to predict the observed seasonality in THMs over the one year monitoring period. Since THM formation was shown to be a function of water temperature and DOC content, THMs (µg/L) can be estimated from those measured a reference point T_x_, DOC_x_ according to

1 $$\begin{array}{c}THMs({T}_{x}+{\rm{\Delta }}T,DO{C}_{x}+{\rm{\Delta }}DOC)\\ \,\approx \,THMs({T}_{x},DO{C}_{x})+\frac{{\rm{\partial }}}{{\rm{\partial }}T}THMs({T}_{x},DO{C}_{x})\ast {\rm{\Delta }}T\\ \,+\,\frac{{\rm{\partial }}}{{\rm{\partial }}DOC}THMs({T}_{x},DO{C}_{x})\ast {\rm{\Delta }}DOC\end{array}$$

T is the average monthly water temperature in °C, and DOC the average monthly total organic carbon concentration in mg/L. To estimate the magnitude of the derivates ∂THMs/∂T and ∂THMs/∂DOC we used the results of the THMFP laboratory experiments. Using the THMFP measured at 1 mg/L DOC and 5 °C as a reference point, there is by linear interpolation between this reference point and the next measured data points an average 4.0 ± 0.2% increase in THMFP per 1 °C increase in temperature towards 15 °C, and an average 66 ± 3% increase in THMFP per 1 mg/L increase in DOC towards 5 mg/L. The laboratory data reference point (5 °C, 1 mg/L DOC), is most closely matched by the case study temperature and DOC data for the month of December (5.75 °C, 1.03 mg/L DOC, 35.4 mg/L THMs). Hence, using the temperature and DOC sensitivities of THM formation measured in the laboratory in combination with the average change in monthly water temperatures and DOC concentrations measured at the five case study sites, the seasonal variation in average THMs (µg/L) for each month was estimated from those measured in December according to:

2 $$\begin{array}{c}THMs(T,DOC)\\ \,=\,35.4+35.4\ast (0.040\pm 0.002)\ast (T-5.75)+35.4\ast (0.66\pm 0.03)\\ \,\ast \,(DOC-1.03)\end{array}$$

The equation assumes that the relative change in THMs concentrations observed in treatment works for variable water temperature and DOC at the point of disinfection is similar to the relative change in THMFP observed in the laboratory chlorination experiments. As shown in Fig. 1a, these estimates closely reproduced the observed seasonal variation in average THM concentrations in potable water of the five case study sites. Hence, THM seasonality can be modelled with a straight-forward approach by considering the sensitivity of THMFP measured in laboratory experiments to variable water temperature and DOC content.

### Climate change scenarios

The UK’s Adaptation Sub-Committee (ASC) has predicted that regional summer mean temperature will increase in Scotland between 0.9–4.5 °C by the 2050 s, of which 0.9 °C may already have occurred^1^. This means that predictions are for a further increase of 0–3.6 °C from current temperatures, or 1.8 °C on average. Evans *et al*.^14^ proposed that 10–20% of the 91% increase in UK surface water DOC over 15 years is explained by the 0.66 °C temperature increase, which equates to a 15.2–30.3% increase in surface water DOC per 1 °C temperature increase, or on average 22.7% per 1 °C. Based on these predictions, we developed a matrix of low/medium/high climate change scenarios, consisting of the various combinations of a 0, 1.8 or 3.6 °C increase in surface water temperature by 2050, and 15.2%, 22.7% or 30.3% increases in surface water DOC per 1 °C increase in surface water temperature. Considering the strong correlations observed in our case study we furthermore assumed that these changes in temperature and surface water DOC will correspond to those occurring in potable water at the point of disinfection.

By combining the climate change predictions with the laboratory data, we estimated the anticipated percent changes in summer THM formation by 2050, if there is no change to the current treatment practice, according to

3 $$\begin{array}{ccc}{\rm{\Delta }}THMs\,({\rm{ \% }}) & = & [\frac{THMs({T}_{x}+{\rm{\Delta }}T,DO{C}_{x}+{\rm{\Delta }}DOC)-THMs({T}_{x},DO{C}_{x})}{THMs({T}_{x},DO{C}_{x})}]\cdot 100{\rm{ \% }}\\  &  & \approx \,[\frac{1}{THMs({T}_{x},DO{C}_{x})}\cdot \frac{{\rm{\partial }}}{{\rm{\partial }}T}THMs({T}_{x},DO{C}_{x})\ast {\rm{\Delta }}T\\  &  & +\,\frac{1}{THMs({T}_{x},DO{C}_{x})}\cdot \frac{{\rm{\partial }}}{{\rm{\partial }}DOC}THMs({T}_{x},DO{C}_{x})\ast {\rm{\Delta }}DOC]\cdot 100{\rm{ \% }}\end{array}$$

We estimated the magnitude of the derivates ∂THMs/∂T and ∂THMs/∂DOC from the laboratory experiments, using the THMFP measured at 1 mg/L DOC and 15 °C as the closest laboratory data reference point for the water quality observed in the summer months in Scotland (i.e. an average temperature of 14.5 °C and 1.3 mg/L DOC at the point of disinfection for June/July/August). The data then suggested a 3.0 ± 0.2% increase in THM formation per 1 °C temperature increase towards 25 °C, and a 63 ± 4% increase in THM formation per 1 mg/L increase in DOC towards 5 mg/L (Fig. 3). We estimated ΔDOC (mg/L) as the anticipated change in potable water DOC (currently 1.3 mg/L) for a ΔT (°C) change in regional mean summer temperature according to

4 $${\rm{\Delta }}DOC=1.3\ast {S}_{1}\ast {\rm{\Delta }}T$$

where 1.3 * S_1_ ((mg/L)/°C) is the DOC response to the temperature increase. Inserting these values into Eq. 3 results in

5 $${\rm{\Delta }}THMs\,( \% )\approx (3.0\pm 0.2\,\frac{ \% }{^\circ C})\ast {\rm{\Delta }}T+(63\pm 4\,\frac{ \% }{mg/L})\ast 1.3\ast {S}_{1}\ast {\rm{\Delta }}T$$

ΔTHMs (%) is the anticipated % change in potable water THMs for a mean summer temperature change of ΔT (0, 1.8 or 3.6 °C for the low, medium and high scenario derived from the ASC predictions), and 1.3 * S_1_ is the DOC response to the temperature increase (1.3 * 0.152, 1.3 * 0.227 or 1.3 * 0.303 ((mg/L)/°C) for the low, medium and high sensitivity scenario derived from Evans *et al*.^14^).

The predicted increases in THM formation for the various scenarios ranged from 0 to 100%, with an increase of 39% being the mid-range scenario (Table 2). The effect of a warming climate on surface water DOC levels is the main contributor to this predicted increase for the mid-range scenario (33.5%), while the direct water temperature effect on THM formation accounts for 5.4%.

**Table 2:** Climate change scenarios and their implications for summer THMs formation by 2050.

	Change in potable water temperature		
Change in potable water TOC	Low ΔT = 0 °C	Medium ΔT = 1.8 °C	High ΔT = 3.6 °C
Low ΔTOC = 15.2%/°C	Increase in THMs formation = 0%	Increase in THMs formation = 28%	Increase in THMs formation = 56%
Medium ΔTOC = 22.7%/°C	Increase in THMs formation = 0%	Increase in THMs formation = 39%	Increase in THMs formation = 78%
High ΔTOC = 30.3%/°C	Increase in THMs formation = 0%	Increase in THMs formation = 50%	Increase in THMs formation = 100%

## Discussion

The monthly monitoring data from five DWTPs over one calendar year confirmed that THM formation is sensitive to seasonal variations, which has implications to climate. This is in line with earlier observations^17,23^, but our monthly monitoring programme was much more comprehensive than earlier studies and comprised 42 parameters of potable and 38 parameters of raw water quality. Nonetheless, amongst the many parameters monitored, only the monthly averages of water temperature, and various indicators of the water organic carbon content had well established statistically significant correlations with the monthly averages of total THMs (Table 1).

To exclude the possibility that these correlations observed in full-scale water treatment data are spurious, we separately investigated, with controlled laboratory experiments, the effects of variations in organic carbon concentration and temperature, and potential seasonal changes in organic carbon quality on THMs formation. From the laboratory test results it was evident that the parameters water temperature and DOC both directly influence THMs formation, as more THMs were consistently formed for a fixed amount of DOC of the same origin, but at higher water temperature, or for a fixed water temperature, but at a higher DOC concentration of the same origin. The findings are in line with earlier literature reports of higher water temperature or higher DOC during disinfection enhancing the formation of by-products in water treatment plants^7,9,24^. In addition, our study excluded from both, monitoring and laboratory data, seasonal DOC quality changes as a major cause for the seasonal variability in THMs, since the DOC quality indicators HPI, TPI and HPO, if expressed as % of DOC, were not correlated with total THMs in the monitoring data, and since in the laboratory experiments, THM formation per mg of DOC was similar for water sampled in different calendar months.

Conducting the laboratory experiments not only firmly established the root causes of seasonal variation in THMs at our case study sites, but also quantified the sensitivity of THMs formation to changes in water temperature and DOC. These temperature and DOC sensitivities of THM formation measured in the laboratory, in combination with the observed changes in average monthly water temperatures and DOC concentrations during the calendar year, enabled us to predict and closely reproduce the seasonal variation in average THM concentrations in potable water observed at the five full-scale DWTPs. The close agreement between predictions and monitoring data gives us confidence in the use of the laboratory data for predicting future climate change related temperature and TOC concentration effects on THMs in full-scale DWTPs.

The increase in regional summer mean temperatures in Scotland predicted by ASC^1^ would inevitably result in higher surface water temperatures. Indeed, an average decadal temperature change in UK river water temperatures of 0.21 ± 0.19 °C has already been noted between 1970 and 2000 as a consequence of an average decadal air temperature change of 0.3 °C, although regional variations are very substantially^25^. This surface water temperature increase would also increase water temperature at the point of disinfection, considering the strong correlation between raw and potable water temperature observed in this study. From our laboratory experiments, higher water temperature would directly promote THMs formation (Fig. 2). In addition, an ambient temperature increase may stimulate soil microbial activity in peatland, and thereby enhance surface water DOC^26^. Other factors such as water levels in peat are also relevant for the microbiological DOC generation^26^. In a detailed analysis of the various potential causes for the 91% increase observed in UK upland water DOC between 1988 and 2003, Evans *et al*.^14^ attributed a DOC increase of 10–20% to a 0.66 °C temperature increase, with the main cause for the overall 91% increase being reduced sulphur deposition. Evans *et al*.^14^ also considered hydrological drivers, and enhanced primary productivity due to raising atmospheric CO_2_, but concluded that these factors could not explain such a significant DOC increase over the observation period. As their report is the most detailed analysis of various factors behind raising DOC in UK surface waters, we used their estimated warming effects in combination with ASC predictions for ambient temperatures in our climate change scenarios. However, there is still no scientific consensus on the causes of the current rise in DOC concentrations, and our results demonstrate a need to ascertain temperature effects on surface water DOC for effective climate change adaptation planning in the water sector.

The uncertainty in the predicted magnitude of the mean summer temperature increase in Scotland^1^ is inevitably reflected in the predicted increases in THM formation, which range from 0 to 100% for the various scenarios (Table 2). The mid-range scenario of a further 39% increase in THM formation would have significant water treatment implications in the British Isles and other regions with peatland in catchments, considering that maintaining THM levels below drinking water quality standards is already a current treatment challenge^7,17^. Since water providers need to maintain water quality standards in a changing climate, further treatment adaptations will likely become necessary to maintain regulatory compliance in the future.

In response to the increasing THM control challenge, Scottish Water has for example over the past few years, already increased monitoring, by more regularly measuring TOC in raw water, and with the installation of online DOC sensors in water treatment works. Scottish Water has also already adapted and optimized the performance of its operations, for example with enhanced coagulation or the careful evaluation and selection of filtration membranes, and with the addition of treatment steps such as air stripping of THMs, granular activated carbon (GAC) filtration or ion exchange. Such treatment upgrades will inevitably have treatment cost implications. An US EPA report^27^ suggests that addition of GAC filtration increases operational water treatment costs by an average 15%, while according to the report, switching from chlorine to UV disinfection (which produces less THMs) would increase cost by about 6%.

Our analysis consequently suggests that environmental change affects surface water quality with implications for water treatment and its costs, as illustrated for the control of trihalomethanes formation in drinking water disinfection. Although this study focused on Scottish systems, implications of temperature change and associated changes in source water DOC are global. As such, equivalent studies are urgently elsewhere in the world to fully understand the health and cost implications of climate change on the world’s potable water supply.

## Methods

### Selection of five case study sites

Case study sites were selected from a total of 93 DWTP sites across Scotland to identify five representative sites. Cluster analysis was performed to achieve this objective using the K-means algorithm by R Studio free software (Integrated Development for R. R Studio, Inc., Boston, MA. Version: 3.3.1). Preliminary exploratory analysis of data involved the division of DWTP sizes into three categories: small, medium and large. Size was defined by applying a histogram of frequency for size (ML/day) using also R Studio software. Minimum size was allocated at 0.006 ML/day and maximum at 110 ML/day. Frequency plots showed that 47 of the DWTPs from the selected 93 sites (51.6%) were small ranging from 0.006 ML/day to 0.838 ML/day, 26 DWTPs (28.6%) were medium size ranging from 0.838 ML/day to 13.5 ML/day and 18 DWTPs (19.8%) were large ranging from 13.5 ML/day to 110 Ml/day. Twenty centroids were used for K-means with a maximum number of 100 iterations. The K groups were plotted against the following quality variables: total THMs (distribution and potable water), TOC (raw water, final potable and distribution networks), colour (raw water), chloride (distribution networks) and bromide (raw water) to determine outliers (anomalies). This approach selected a preliminary set of 21 DWTPs that were grouped per region from which the final selection of five case study sites was narrowed down according to accessibility, type of treatment (conventional coagulation and membrane filtration) and geographical location. The five sites are described in Table S1 in Supplementary Information.

### Sampling monitoring strategy

Two sampling strategies were applied during this study to the five case study sites. One strategy collected samples every month from December 2014 to November 2015 and analytical work was performed by Scottish Water Scientific Services (SWSS) for raw water samples and final potable water (i.e. treated) samples. Analysis by SWSS followed a scheduled sampling programme and certified analytical protocols approved by the Drinking Water Quality Regulator (DWQR) for Scotland and the United Kingdom Accreditation Service (UKAS)^28^. All data were extracted and processed for statistical analysis from the Scottish Water Laboratory Information Management System (LIMS). Potable water parameters comprised ammonium, chloride, colour, conductivity, fluoride, pH, nitrate, nitrite, soluble reactive phosphate, sulphate, total organic nitrogen ratio (TON.ratio), total organic carbon (TOC), total organic nitrogen (TON), light transmittance at 220 nm, 254 nm, 270 nm, 350 nm, turbidity, bromide, bromodichloromethane, bromoform, chlorate, chloroform, dibromochloromethane, total trihalomethanes, free chlorine, temperature, total chlorine, aluminium, calcium, copper, iron, lead, magnesium, manganese, phosphorus, potassium, sodium, zinc, the hydrophobic organic fraction (HPO), transphilic organic fraction (TPI), and hydrophilic organic fraction (HPI) of TOC. Raw water parameters comprised alkalinity, ammonium, chloride, colour, conductivity, fluoride, pH, nitrate, nitrite, soluble reactive phosphate, sulphate, TON.ratio, TOC filtered, TOC, TON, light transmittance at 220 nm, 254 nm, 270 nm, 350 nm, UV transmittance, turbidity, bromide, temperature, aluminium, calcium, copper, iron, lead, magnesium, manganese, phosphorus, potassium, sodium, zinc, chlorophyll, the hydrophobic organic fraction (HPO), transphilic organic fraction (TPI), and hydrophilic organic fraction (HPI) of TOC. THMs were measured using a modified in house method based on EPA Method 524.2 for purgeable organic compounds in water by capillary column gas chromatography mass spectrometry. Fractional separation of Natural Organic Matter (NOM) into Hydrophobic (HPO), Transphilic (TPI) and Hydrophilic (HPI) fractions was performed by filtering water samples through a 0.45 µm nylon membrane (Millipore, Darmstad, Germany) before pumping the water through a series of chromatographic columns containing Amberlite® XAD 4 and XAD 7 resins (Sigma Aldrich, Darmstad, Germany) for the fractional separation. Each fraction was eluted from the resins using an alkali solution (0.1 M NaOH) and rinsed with an acidic solution (0.1 M HCl). A Total Organic Carbon analyser (Shimadzu, Kyoto, Japan) was used to determine the concentration of the DOC in each fraction^29^. Measured potable and raw water characteristics for each calendar month were compiled Tables S2 and S3 as Supporting Information.

The second sampling strategy collected samples quarterly in 5 L high density polyethylene containers with caps purchased from VWR, Poole, England. Samples were delivered to the School of Engineering (Newcastle University) where samples were filtered through glass fibre pre filters (Millipore, Cork, Ireland) to remove larger particulates and then through 0.45 µm polyamide membranes (Sartorius, Goettingen, Germany) to be stored at 4 °C until for further use. These samples were used for THMFP measurements.

### Trihalomethanes formation potential (THMFP)

Ammonium chloride, sodium hydroxide, sodium thiosulphate, sodium chloride, potassium bromide, potassium chloride, aluminium oxide, mono and dipotassium phosphate, disodium hydrogen phosphate, calcium carbonate, sodium bicarbonate, iron sulphate heptahydrate, sodium hypochlorite (12%) and manganese sulphate hydrate salts were purchased from BDH (Poole, England) with 99% purity. Sodium sulphate, magnesium carbonate, orthophosphoric acid, pentane (pesticide residue analysis grade) and 1,2-dibromopropane were purchased from Sigma-Aldrich (St. Louis, USA) with 99% purity. A trihalomethanes calibration mix with 2000 µg/mL for each compound in methanol was purchased from Supelco (Bellefonte, USA). DOC concentrations were measured in each raw and treated water samples from each case study site in order to be able to dilute them, if necessary, to either 5 mg DOC/L (raw water only) or 1 mg DOC/L, with a salt solution that mimicked typical Scottish raw water composition (Table S7 in Supporting Information). DOC was determined using a total organic carbon analyser TOC-5050A coupled with an autosampler ASI-5000A (Shimadzu, Japan).

Water solutions with DOC content of 1 mg/L or 5 mg/L (270 mL) where transferred into amber glass bottles with glass caps and buffered with 2.74 mL phosphate buffer solution (34 g KHPO_4_ and 5.85 g NaOH in 250 mL deionised water) and immediately chlorinated with 1.4 mL of a 1000 mg/L sodium hypochlorite solution previously standardised with 0.1 M sodium thiosulphate according to APHA methods and filled to the brim with deionised water to prevent any oxygen from entering the reaction vessel. Each sample was prepared in triplicate and incubated at 5 °C, 15 °C and 25 °C. After seven days (+/−4 hours), THMs were liquid-liquid extracted and analyzed following a previously described method protocol (Werner *et al*. 2016).

### Data evaluation and statistical methods

Of the 42 potable water quality parameters studied in the yearly monitoring programme, ten parameters (ammonium, colour, nitrate, nitrite, TON.ratio, TON, turbidity, iron, lead, manganese) had >50% below detection limit values and were excluded from the correlation analysis. Linear correlation coefficients and corresponding p-values for testing the hypothesis of no correlation against the alternative of a nonzero correlation were then calculated for the remaining parameters using Matlab R2016a ©. To make the seasonal variation of different parameters more readily comparable in a single plot, the monitoring data is displayed in figures as the monthly mean for the five case study sites divided by the mean parameter value for the entire observation period, and the uncertainty is indicated by error bars equal to plus and minus one standard error of the monthly mean divided by the mean parameter value for the entire observation period. THM formation experiments for different conditions were performed in triplicates. Mean values were then calculated for different conditions such as DOC values, temperatures, etc., from the mean value of the triplicate measurements. Experimental outcomes were reported/displayed as the mean plus minus one standard error of the mean. Two-tailed z-tests using Excel 2016 © were used to test the hypothesis of no change in THM formation against the alternative of a nonzero percent change. Two-tailed Welch tests (or unequal variances t-test) using Excel 2016 © were used to test the hypothesis of equal means for the two data sets against the alternative of a nonzero difference in the mean.

## Supplementary information

Supplementary information

## Data Availability

All data created during this research are openly available at DOI 10.25405/data.ncl.8325392. Please contact Newcastle Research Data Service at rdm@ncl.ac.uk for access instructions.
