# Supplementary material for: Predicted Impact of Climate Change on Trihalomethanes Formation in Drinking Water Treatment
Source: Sci Rep. 2019 Jul 10;9:9967. doi: 10.1038/s41598-019-46238-0 (PMC6620267; doi:10.1038/s41598-019-46238-0)

**SUPPLEMENTARY INFORMATION**

**PREDICTED IMPACT OF CLIMATE CHANGE ON TRIHALOMETHANES FORMATION IN DRINKING WATER TREATMENT**

Maria Valdivia-Garcia^†§^, Paul Weir^§^, David W. Graham^§^, and David Werner^†^*

^†^ School of Engineering

Newcastle University, Newcastle upon Tyne, United Kingdom

^§^Scottish Water, Castle House, Dunfermline, Edinburgh, United Kingdom

*To whom correspondence should be addressed.

E-mail: david.werner@ncl.ac.uk

Phone: (0044) 191 208 5099

**Table S1:** Five study case sites description. RGF: rapid gravity filtration; GAC: granulated activated carbon; PF: pressure filtration; DAF: dissolved air flotation

| **Site ID** | **Size** | **Treatment Type** | **Disinfection** | **Soil Type** | **Sampling points** |
| --- | --- | --- | --- | --- | --- |
|  | **(ML/day)** |  |  |  |  |
|  |  |  |  |  |  |
| **AWTP** | 20 | Coagulation/ | Chlorination | Shallow peat | Raw water |
|  |  | DAF/PF |  |  |  |
|  |  |  |  |  | Post sand filtration |
|  |  |  |  |  |  |
|  |  |  |  |  |  |
| **BWTP** | 0.2 | Tubular Membrane/ | Chlorination | Peaty gley | Raw water |
|  |  | GAC filtration |  |  |  |
|  |  |  |  |  | Post GAC filtration |
|  |  |  |  |  |  |
| **GWTP** | 0.3 | Tubular Membranes/ | Chlorination | Shallow peat | Raw water |
|  |  | GAC filtration |  |  |  |
|  |  |  |  |  | Post GAC filtration |
|  |  |  |  |  |  |
| **FWTP** | 20 | Coagulation/ | Chlorination | Brown forest soils | Raw water |
|  |  | RGF/GAC filtration |  | with gleying |  |
|  |  |  |  |  |  |
|  |  |  |  |  | Post GAC filtration |
|  |  |  |  |  |  |
| **SWTP** | 6.5 | Coagulation/RGF | Chlorination | Boggy peat/ | Raw water |
|  |  |  |  | brown forests with gleying |  |
|  |  |  |  |  | Post sand filtration |
|  |  |  |  |  |  |

**Table S2:** Potable water quality for the five case study sites, mean and standard error for each month, n.a. = not available

|  | Chloride | Conductivity | Fluoride | pH | Phosphate (sol.) | Sulphate | TOC | T220 | T254 | T270 | T350 | Chlorate | Bromide |
| --- | --- | --- | --- | --- | --- | --- | --- | --- | --- | --- | --- | --- | --- |
|  | mg/L | µS/cm | mg/L |  | µg/L | mg/L | mg/L | % | % | % | % | µg/L | µg/L |
| Mean | | | | | | | | | | | | | |
| Jan | 40.400 | 221.800 | 0.047 | 7.940 | 163.800 | 28.680 | 0.840 | 68.828 | 95.272 | 96.420 | 99.304 | 56.080 | 49.200 |
| Feb | 36.000 | 209.750 | 0.056 | 8.150 | 96.750 | 29.450 | 0.700 | 66.720 | 96.108 | 97.100 | 98.865 | 38.300 | 37.300 |
| Mar | 39.000 | 207.800 | 0.052 | 8.120 | 127.400 | 24.220 | 0.600 | 74.374 | 97.134 | 97.866 | 99.724 | 52.050 | 53.325 |
| Apr | 35.000 | 196.400 | 0.054 | 7.880 | 133.800 | 24.360 | 0.740 | 74.364 | 96.712 | 97.606 | 99.512 | 62.920 | 41.920 |
| May | 34.000 | 197.800 | 0.051 | 8.100 | 157.000 | 24.740 | 0.960 | 73.398 | 95.650 | 96.748 | 99.328 | 86.120 | 30.620 |
| Jun | 32.800 | 202.200 | 0.105 | 8.160 | 184.000 | 25.100 | 1.040 | 70.984 | 94.920 | 96.316 | 99.056 | 158.367 | 30.400 |
| Jul | 41.000 | 186.500 | 0.047 | 7.700 | 74.500 | 12.550 | 1.300 | 88.845 | 94.490 | 96.365 | 99.195 | 190.650 | 39.150 |
| Aug | 29.200 | 201.200 | 0.072 | 8.140 | 154.600 | 31.960 | 1.560 | 69.348 | 93.496 | 95.148 | 99.028 | 130.325 | 27.450 |
| Sep | 29.200 | 209.200 | 0.073 | 8.080 | 153.600 | 33.140 | 1.460 | 68.344 | 93.354 | 94.914 | 98.870 | 124.350 | 65.750 |
| Oct | 29.750 | 218.000 | 0.054 | 8.275 | 190.750 | 35.325 | 1.425 | 63.973 | 93.280 | 95.070 | 98.973 | 97.700 | 31.100 |
| Nov | 24.800 | 184.400 | 0.054 | 7.725 | 151.200 | 31.820 | 1.280 | 68.720 | 94.324 | 96.094 | 99.330 | n.a. | n.a |
| Dec | 33.500 | 223.500 | 0.042 | 7.775 | 140.250 | 36.500 | 1.025 | 63.323 | 93.938 | 95.103 | 98.645 | n.a | n.a |
| Standard Error | | | | | | | | | | | | | |
| Jan | 9.395 | 38.099 | 0.005 | 0.252 | 68.005 | 10.909 | 0.172 | 16.329 | 0.752 | 0.647 | 0.133 | 19.827 | 16.777 |
| Feb | 10.512 | 48.871 | 0.009 | 0.155 | 84.762 | 11.490 | 0.147 | 21.054 | 1.171 | 0.967 | 0.950 | 17.443 | 17.200 |
| Mar | 7.190 | 33.996 | 0.007 | 0.242 | 68.734 | 10.178 | 0.138 | 17.735 | 0.555 | 0.356 | 0.120 | 22.424 | 14.627 |
| Apr | 8.037 | 40.598 | 0.008 | 0.120 | 74.252 | 11.064 | 0.087 | 17.492 | 0.482 | 0.351 | 0.107 | 36.952 | 13.766 |
| May | 8.087 | 33.940 | 0.004 | 0.145 | 66.236 | 10.934 | 0.136 | 16.517 | 0.533 | 0.437 | 0.178 | 37.106 | 11.859 |
| Jun | 8.540 | 35.787 | 0.047 | 0.236 | 83.121 | 11.063 | 0.214 | 16.400 | 0.916 | 0.703 | 0.217 | 64.709 | 17.610 |
| Jul | 15.000 | 25.500 | 0.005 | 0.100 | 64.500 | 10.450 | 0.400 | 1.795 | 1.330 | 1.095 | 0.255 | 0.250 | 26.350 |
| Aug | 8.726 | 38.443 | 0.019 | 0.242 | 85.933 | 14.440 | 0.357 | 14.466 | 1.443 | 1.190 | 0.405 | 60.634 | 16.504 |
| Sep | 8.817 | 34.620 | 0.019 | 0.256 | 75.425 | 14.740 | 0.220 | 14.692 | 1.154 | 0.926 | 0.277 | 87.250 | 19.450 |
| Oct | 10.355 | 44.319 | 0.008 | 0.217 | 74.130 | 19.166 | 0.259 | 19.051 | 1.821 | 1.502 | 0.470 | 86.700 | 24.100 |
| Nov | 7.338 | 33.563 | 0.010 | 0.075 | 67.491 | 13.389 | 0.248 | 15.864 | 0.601 | 0.536 | 0.150 | n.a | n.a |
| Dec | 9.438 | 47.596 | 0.000 | 0.232 | 63.875 | 13.069 | 0.427 | 20.387 | 1.746 | 1.463 | 0.402 | n.a | n.a |

**Table S2 continued:** Potable water quality for the five case study sites, mean and standard error for each month, n.a. = not available

|  | Chlorine (free) | Chlorine (tot) | Temp. | Aluminum | Calcium | Copper | Magnesium | Phosphorus | Potassium | Sodium | Zinc | HPI | HPO | TPI |
| --- | --- | --- | --- | --- | --- | --- | --- | --- | --- | --- | --- | --- | --- | --- |
|  | mg/L | mg/L | °C | mg/L | mg/L | mg/L | mg/L | µgP/L | mg/L | mg/L | µg/L | mg | mg | mg |
| Mean | | | | | | | | | | | | | | |
| Jan | 0.564 | 0.652 | 3.880 | 23.800 | 15.300 | 0.004 | 3.880 | 163.400 | 1.560 | 24.600 | 4.800 | 0.346 | 0.412 | 0.181 |
| Feb | 0.605 | 0.688 | 4.150 | 19.500 | 15.175 | 0.003 | 3.850 | 124.750 | 1.275 | 22.475 | 3.500 | 0.231 | 0.600 | 0.169 |
| Mar | 0.548 | 0.610 | 6.225 | 14.600 | 14.640 | 0.003 | 4.040 | 136.400 | 1.420 | 22.980 | 3.400 | 0.314 | 0.319 | 0.127 |
| Apr | 0.592 | 0.648 | 9.940 | 22.800 | 14.400 | 0.004 | 3.560 | 154.600 | 1.300 | 21.400 | 5.600 | 0.458 | 0.340 | 0.162 |
| May | 0.520 | 0.622 | 11.280 | 23.400 | 14.860 | 0.003 | 3.900 | 177.800 | 1.200 | 20.820 | 5.000 | 0.449 | 0.406 | 0.206 |
| Jun | 0.536 | 0.612 | 13.600 | 24.000 | 16.060 | 0.004 | 4.260 | 208.000 | 1.100 | 21.560 | 4.800 | 0.483 | 0.476 | 0.281 |
| Jul | 0.450 | 0.510 | 14.300 | 25.500 | 6.550 | 0.002 | 2.800 | 102.000 | 1.050 | 31.600 | 6.000 | 0.513 | 0.592 | 0.245 |
| Aug | 0.548 | 0.622 | 15.520 | 22.600 | 17.440 | 0.003 | 3.700 | 177.200 | 1.260 | 20.780 | 3.000 | 0.555 | 0.620 | 0.286 |
| Sep | 0.500 | 0.590 | 12.920 | 17.400 | 18.380 | 0.003 | 4.140 | 177.200 | 1.160 | 21.120 | 4.000 | 0.826 | 0.637 | 0.317 |
| Oct | 0.553 | 0.638 | 10.125 | 17.750 | 22.575 | 0.004 | 4.375 | 205.500 | 1.425 | 17.125 | 5.000 | 0.643 | 0.531 | 0.300 |
| Nov | 0.720 | 0.742 | 8.260 | 26.400 | 16.340 | 0.003 | 3.020 | 167.600 | 1.620 | 19.040 | 5.000 | 0.527 | 0.623 | 0.250 |
| Dec | 0.513 | 0.578 | 5.750 | 29.250 | 16.700 | 0.003 | 3.450 | 140.250 | 1.825 | 25.325 | 3.250 | 0.634 | 0.598 | 0.218 |
| Standard error | | | | | | | | | | | | | | |
| Jan | 0.080 | 0.074 | 0.892 | 9.030 | 5.471 | 0.002 | 0.802 | 67.542 | 0.614 | 6.237 | 1.594 | 0.053 | 0.070 | 0.037 |
| Feb | 0.077 | 0.089 | 0.880 | 6.397 | 6.052 | 0.001 | 1.090 | 74.750 | 0.650 | 8.213 | 1.500 | 0.106 | 0.124 | 0.056 |
| Mar | 0.038 | 0.043 | 0.592 | 4.456 | 5.037 | 0.002 | 0.900 | 66.162 | 0.511 | 5.111 | 0.748 | 0.049 | 0.072 | 0.036 |
| Apr | 0.077 | 0.082 | 0.990 | 7.513 | 5.154 | 0.001 | 0.981 | 67.418 | 0.472 | 4.924 | 1.749 | 0.035 | 0.046 | 0.034 |
| May | 0.097 | 0.074 | 0.910 | 7.672 | 4.800 | 0.001 | 0.970 | 59.165 | 0.462 | 4.862 | 1.673 | 0.084 | 0.069 | 0.027 |
| Jun | 0.041 | 0.039 | 0.855 | 9.349 | 5.070 | 0.002 | 1.166 | 77.436 | 0.422 | 5.422 | 1.241 | 0.089 | 0.100 | 0.047 |
| Jul | 0.050 | 0.080 | 1.300 | 16.500 | 5.750 | 0.001 | 1.600 | 52.000 | 0.650 | 2.800 | 4.000 | 0.111 | 0.208 | 0.031 |
| Aug | 0.076 | 0.076 | 1.176 | 8.183 | 6.063 | 0.001 | 0.988 | 84.437 | 0.644 | 5.831 | 0.775 | 0.097 | 0.103 | 0.062 |
| Sep | 0.078 | 0.085 | 0.348 | 7.672 | 5.839 | 0.001 | 1.115 | 69.293 | 0.581 | 5.765 | 1.140 | 0.129 | 0.130 | 0.068 |
| Oct | 0.087 | 0.088 | 0.778 | 8.420 | 5.737 | 0.001 | 0.936 | 63.604 | 0.791 | 5.794 | 1.225 | 0.136 | 0.083 | 0.044 |
| Nov | 0.067 | 0.066 | 0.303 | 9.714 | 5.981 | 0.001 | 0.795 | 62.831 | 0.784 | 5.713 | 1.673 | 0.084 | 0.132 | 0.044 |
| Dec | 0.052 | 0.059 | 1.148 | 9.911 | 7.288 | 0.002 | 1.005 | 63.407 | 0.749 | 7.649 | 1.250 | 0.108 | 0.252 | 0.085 |

**Table S2 continued:** Potable water quality for the five case study sites, mean and standard error for each month, n.a. = not available

|  | Bromodichloro- methane | Bromoform | Chloroform | Dibromochloro- methane | THMs |
| --- | --- | --- | --- | --- | --- |
|  | µg/L | µg/L | µg/L | µg/L | µg/L |
| Mean | | | | | |
| Jan | 6.740 | 5.660 | 12.380 | 8.700 | 33.020 |
| Feb | 6.250 | 5.675 | 14.400 | 6.650 | 32.325 |
| Mar | 5.220 | 7.140 | 7.940 | 8.760 | 28.620 |
| Apr | 7.540 | 5.660 | 13.360 | 8.540 | 34.540 |
| May | 8.660 | 5.320 | 20.260 | 8.800 | 42.400 |
| Jun | 9.040 | 4.960 | 23.720 | 8.680 | 45.740 |
| Jul | 13.450 | 6.150 | 24.850 | 14.350 | 58.550 |
| Aug | 10.060 | 5.160 | 34.520 | 8.780 | 57.900 |
| Sep | 12.060 | 4.040 | 37.740 | 9.200 | 62.300 |
| Oct | 8.250 | 6.950 | 34.200 | 9.750 | 58.475 |
| Nov | 7.220 | 3.320 | 22.000 | 5.960 | 37.840 |
| Dec | 5.750 | 4.950 | 17.825 | 7.500 | 35.425 |
| Standard error | | | | | |
| Jan | 1.636 | 2.998 | 5.158 | 3.146 | 4.389 |
| Feb | 1.716 | 3.206 | 6.814 | 3.360 | 4.515 |
| Mar | 1.046 | 2.468 | 4.044 | 2.630 | 3.206 |
| Apr | 1.737 | 3.114 | 5.179 | 3.832 | 4.685 |
| May | 1.950 | 2.957 | 7.199 | 4.354 | 5.052 |
| Jun | 1.899 | 2.739 | 8.744 | 4.621 | 6.502 |
| Jul | 0.550 | 5.650 | 19.350 | 10.750 | 3.250 |
| Aug | 2.436 | 2.859 | 12.708 | 4.995 | 8.315 |
| Sep | 3.617 | 2.181 | 12.147 | 5.395 | 11.144 |
| Oct | 2.332 | 3.735 | 17.941 | 6.101 | 11.758 |
| Nov | 1.773 | 1.733 | 7.996 | 3.205 | 6.050 |
| Dec | 1.732 | 2.508 | 12.408 | 3.476 | 8.957 |

**Table S3:** Raw water quality for the five case study sites, mean and standard error for each month, n.a. = not available

|  | Alkalinity | Ammonium | Chloride | Colour | Conductivity | Fluoride | pH | Nitrate | Nitrite | Phosphate (sol.) | Sulphate | TON.ratio | TOC (filtered) |
| --- | --- | --- | --- | --- | --- | --- | --- | --- | --- | --- | --- | --- | --- |
|  | mgCaCO3/L | mg/L | mg/L | Pt-Co | µS/cm | mg/L |  | mg/L | mg/L | µg/L | mg/L | 0 | mg/L |
| Mean | | | | | | | | | | | | | |
| Jan | 25.000 | 0.030 | 39.000 | 55.400 | 169.400 | 0.078 | 6.980 | 5.374 | 0.012 | 68.200 | 8.840 | 0.108 | 5.540 |
| Feb | 23.600 | 0.030 | 39.400 | 41.600 | 171.200 | 0.066 | 7.020 | 5.410 | 0.014 | 31.600 | 8.500 | 0.110 | 4.640 |
| Mar | 25.200 | 0.032 | 37.000 | 31.200 | 167.800 | 0.082 | 7.080 | 5.624 | 0.016 | 14.000 | 8.120 | 0.116 | 4.380 |
| Apr | 26.800 | 0.032 | 34.000 | 33.600 | 156.400 | 0.053 | 7.220 | 4.688 | 0.016 | 72.600 | 7.400 | 0.096 | 4.550 |
| May | 29.000 | 0.036 | 32.800 | 48.200 | 149.800 | 0.050 | 7.120 | 4.408 | 0.019 | 38.600 | 6.580 | 0.092 | 5.880 |
| Jun | 30.800 | 0.030 | 30.400 | 52.200 | 147.400 | 0.053 | 7.120 | 4.912 | 0.015 | 42.600 | 6.300 | 0.100 | 6.260 |
| Jul | 25.000 | 0.040 | 38.500 | 80.000 | 145.500 | 0.047 | 6.850 | 1.000 | 0.010 | 150.000 | 2.700 | 0.020 | 8.750 |
| Aug | 32.000 | 0.038 | 27.000 | 99.200 | 132.600 | 0.069 | 7.060 | 3.246 | 0.018 | 53.400 | 5.580 | 0.068 | 10.640 |
| Sep | 37.800 | 0.036 | 27.400 | 97.000 | 141.000 | 0.282 | 7.120 | 4.156 | 0.018 | 79.000 | 5.920 | 0.086 | 9.660 |
| Oct | 31.200 | 0.030 | 26.200 | 92.600 | 134.600 | 0.054 | 7.320 | 4.038 | 0.016 | 61.600 | 3.880 | 0.084 | 10.840 |
| Nov | 28.800 | 0.038 | 22.600 | 71.200 | 122.200 | 0.056 | 6.960 | 4.620 | 0.014 | 84.200 | 6.740 | 0.094 | 10.940 |
| Dec | 23.400 | 0.030 | 31.200 | 76.800 | 145.600 | 0.032 | 7.000 | 5.304 | 0.019 | 72.000 | 7.660 | 0.110 | 8.520 |
| Standard error | | | | | | | | | | | | | |
| Jan | 8.497 | 0.000 | 9.955 | 23.376 | 42.669 | 0.029 | 0.353 | 4.367 | 0.002 | 16.045 | 2.954 | 0.088 | 2.431 |
| Feb | 7.679 | 0.000 | 9.325 | 19.554 | 39.581 | 0.007 | 0.364 | 4.410 | 0.003 | 13.321 | 2.618 | 0.090 | 1.395 |
| Mar | 8.737 | 0.002 | 7.681 | 11.285 | 36.888 | 0.024 | 0.337 | 4.434 | 0.006 | 4.000 | 2.667 | 0.091 | 1.091 |
| Apr | 9.324 | 0.002 | 7.752 | 13.254 | 40.732 | 0.008 | 0.425 | 3.688 | 0.006 | 62.600 | 2.847 | 0.076 | 1.434 |
| May | 9.257 | 0.006 | 8.576 | 14.810 | 40.207 | 0.004 | 0.325 | 3.408 | 0.009 | 26.172 | 2.817 | 0.072 | 1.046 |
| Jun | 10.437 | 0.000 | 8.761 | 15.111 | 42.184 | 0.007 | 0.350 | 3.912 | 0.005 | 25.309 | 3.060 | 0.080 | 1.052 |
| Jul | 20.000 | 0.010 | 18.500 | 47.000 | 80.500 | 0.005 | 0.650 | 0.000 | 0.000 | 140.000 | 0.800 | 0.000 | 1.450 |
| Aug | 10.252 | 0.008 | 9.402 | 20.937 | 41.662 | 0.017 | 0.284 | 2.246 | 0.008 | 33.160 | 3.138 | 0.048 | 1.946 |
| Sep | 11.847 | 0.006 | 8.880 | 26.012 | 42.354 | 0.218 | 0.302 | 3.156 | 0.008 | 55.577 | 3.253 | 0.066 | 2.006 |
| Oct | 10.087 | 0.000 | 8.071 | 27.609 | 41.093 | 0.008 | 0.185 | 3.038 | 0.006 | 38.190 | 1.224 | 0.064 | 2.128 |
| Nov | 9.967 | 0.008 | 8.358 | 27.319 | 38.657 | 0.011 | 0.314 | 3.620 | 0.004 | 23.913 | 2.891 | 0.074 | 2.465 |
| Dec | 7.104 | 0.000 | 7.546 | 28.997 | 39.661 | 0.010 | 0.315 | 4.304 | 0.009 | 18.918 | 2.666 | 0.090 | 2.653 |

**Table S3 continued:** Raw water quality for the five case study sites, mean and standard error for each month, n.a. = not available

|  | TOC | TON | T220 | T254 | T270 | T350 | Turbidity | UV transmittance | Bromide | Temp. | Aluminium | Calcium | Copper |
| --- | --- | --- | --- | --- | --- | --- | --- | --- | --- | --- | --- | --- | --- |
|  | mg/L | mg/L | % | % | % | % | NTU | % | µg/L | °C | µg/L | mg/L | mg/L |
| Mean | | | | | | | | | | | | | |
| Jan | 6.980 | 5.380 | 32.314 | 53.738 | 57.522 | 81.112 | 12.740 | 50.233 | 94.620 | 5.060 | 620.000 | 9.960 | 0.013 |
| Feb | 5.240 | 5.418 | 39.390 | 61.764 | 65.932 | 85.322 | 2.420 | 51.700 | 96.500 | 5.040 | 95.000 | 8.800 | 0.006 |
| Mar | 4.680 | 5.634 | 42.548 | 64.786 | 68.728 | 87.068 | 2.200 | 64.375 | 90.180 | 6.240 | 48.000 | 9.880 | 0.006 |
| Apr | 4.800 | 4.698 | 40.228 | 63.912 | 67.858 | 86.910 | 1.260 | 62.760 | 82.060 | 10.900 | 32.200 | 9.340 | 0.007 |
| May | 6.200 | 4.424 | 33.936 | 54.438 | 58.892 | 82.306 | 1.060 | 54.220 | 71.180 | 11.525 | 38.600 | 9.600 | 0.004 |
| Jun | 6.460 | 4.922 | 27.334 | 50.964 | 55.332 | 79.872 | 0.880 | 51.220 | 95.800 | 13.480 | 47.200 | 10.200 | 0.004 |
| Jul | 8.950 | 1.000 | 24.850 | 38.360 | 43.025 | 72.735 | 1.350 | 38.650 | 122.000 | 14.350 | 36.000 | 7.650 | 0.006 |
| Aug | 11.460 | 3.260 | 12.930 | 27.544 | 31.858 | 63.282 | 1.920 | 19.000 | 81.340 | 14.680 | 73.000 | 9.680 | 0.004 |
| Sep | 13.620 | 4.170 | 19.706 | 36.784 | 40.978 | 70.216 | 5.040 | 36.833 | 100.660 | 12.420 | 88.800 | 10.640 | 0.006 |
| Oct | 10.780 | 4.048 | 28.624 | 42.028 | 45.816 | 71.388 | 3.200 | n.a. | 80.520 | 9.720 | 122.000 | 6.440 | 0.003 |
| Nov | 14.680 | 4.628 | 19.162 | 35.306 | 39.746 | 68.304 | 10.860 | 35.100 | 68.000 | 7.820 | 192.400 | 9.820 | 0.005 |
| Dec | 10.480 | 5.318 | 21.620 | 43.224 | 44.518 | 73.888 | 2.780 | 42.840 | 76.033 | 5.300 | 278.600 | 8.600 | 0.005 |
| Standard error | | | | | | | | | | | | | |
| Jan | 2.187 | 4.373 | 12.989 | 10.660 | 10.640 | 6.499 | 11.644 | 19.150 | 30.761 | 0.800 | 568.749 | 3.526 | 0.009 |
| Feb | 1.631 | 4.418 | 13.576 | 9.704 | 9.573 | 5.530 | 1.026 | 26.200 | 30.540 | 0.664 | 53.029 | 3.014 | 0.004 |
| Mar | 1.087 | 4.444 | 13.966 | 8.630 | 8.208 | 4.449 | 0.846 | 11.178 | 25.939 | 0.733 | 19.398 | 3.436 | 0.004 |
| Apr | 1.371 | 3.698 | 12.337 | 7.656 | 7.388 | 4.189 | 0.286 | 7.676 | 27.322 | 0.996 | 10.613 | 3.384 | 0.005 |
| May | 1.144 | 3.424 | 10.850 | 7.321 | 7.125 | 4.303 | 0.280 | 7.484 | 26.097 | 1.188 | 17.337 | 3.420 | 0.002 |
| Jun | 1.056 | 3.922 | 8.977 | 7.843 | 7.820 | 5.152 | 0.153 | 7.979 | 32.973 | 0.795 | 20.929 | 3.622 | 0.002 |
| Jul | 1.850 | 0.000 | 12.790 | 14.830 | 15.295 | 12.135 | 0.050 | 15.250 | 65.100 | 0.850 | 27.000 | 6.950 | 0.004 |
| Aug | 2.111 | 2.260 | 5.104 | 5.695 | 5.894 | 4.622 | 0.547 | 3.740 | 30.392 | 0.814 | 20.993 | 3.369 | 0.001 |
| Sep | 4.620 | 3.170 | 9.074 | 9.494 | 9.655 | 7.784 | 3.342 | 9.763 | 30.601 | 0.488 | 34.327 | 3.642 | 0.003 |
| Oct | 2.103 | 3.048 | 16.014 | 15.405 | 15.185 | 10.028 | 1.851 | n.a. | 30.381 | 0.348 | 62.917 | 2.934 | 0.001 |
| Nov | 4.654 | 3.628 | 9.183 | 9.356 | 9.556 | 7.350 | 9.762 | 9.421 | 24.859 | 0.511 | 132.976 | 3.544 | 0.003 |
| Dec | 2.953 | 4.318 | 8.736 | 10.028 | 8.596 | 7.929 | 1.454 | 10.133 | 39.889 | 0.572 | 216.521 | 2.959 | 0.003 |

**Table S3 continued:** Raw water quality for the five case study sites, mean and standard error for each month, n.a. = not available

|  | Lead | Magnesium | Manganese | Phosphorus | Potassium | Sodium | Zinc | Chlorophyll | HPI | HPO | TPI |
| --- | --- | --- | --- | --- | --- | --- | --- | --- | --- | --- | --- |
|  | µg/L | mg/L | µg/L | µgP/L | mg/L | mg/L | µg/L | µg/L | mg | mg | mg |
| Mean | | | | | | | | | | | |
| Jan | 2.000 | 3.980 | 257.680 | 273.800 | 1.800 | 20.180 | 21.600 | 7.352 | 0.730 | 4.701 | 1.286 |
| Feb | 0.540 | 3.840 | 26.180 | 113.200 | 1.640 | 20.840 | 9.000 | 3.932 | 0.883 | 4.128 | 0.929 |
| Mar | 0.480 | 3.780 | 18.680 | 50.800 | 1.500 | 19.560 | 8.200 | 7.346 | 0.644 | 3.008 | 0.749 |
| Apr | 0.460 | 3.500 | 32.160 | 195.000 | 1.360 | 18.620 | 7.000 | 13.864 | 0.842 | 3.031 | 0.887 |
| May | 0.420 | 3.340 | 18.420 | 134.000 | 1.220 | 17.140 | 6.000 | 4.294 | 0.949 | 4.021 | 1.210 |
| Jun | 0.400 | 3.440 | 27.500 | 138.600 | 1.140 | 17.180 | 5.400 | 2.902 | 0.923 | 4.373 | 1.265 |
| Jul | 0.400 | 3.150 | 41.100 | 252.000 | 1.150 | 20.550 | 5.500 | 11.215 | 1.438 | 5.469 | 1.543 |
| Aug | 0.460 | 3.140 | 40.540 | 137.200 | 1.240 | 14.920 | 6.800 | 5.016 | 1.481 | 7.816 | 2.002 |
| Sep | 0.940 | 3.360 | 89.280 | 139.200 | 1.220 | 15.460 | 26.000 | 4.726 | 1.579 | 6.550 | 1.891 |
| Oct | 0.520 | 2.200 | 22.260 | 157.200 | 0.620 | 11.420 | 4.600 | 3.726 | 1.639 | 6.460 | 2.121 |
| Nov | 0.860 | 3.160 | 354.700 | 302.800 | 1.900 | 13.440 | 7.200 | 8.640 | 1.694 | 6.661 | 1.885 |
| Dec | 1.160 | 3.360 | 75.120 | 199.600 | 1.740 | 17.380 | 8.600 | 3.480 | 1.817 | 6.019 | 1.402 |
| Standard error | | | | | | | | | | | |
| Jan | 1.297 | 1.016 | 244.914 | 137.126 | 0.736 | 5.322 | 10.515 | 4.837 | 0.152 | 1.854 | 0.243 |
| Feb | 0.172 | 0.929 | 7.979 | 58.803 | 0.622 | 4.762 | 4.494 | 1.473 | 0.237 | 1.118 | 0.248 |
| Mar | 0.196 | 0.910 | 5.615 | 0.800 | 0.573 | 4.143 | 4.017 | 2.953 | 0.081 | 0.904 | 0.217 |
| Apr | 0.166 | 1.046 | 14.924 | 145.000 | 0.487 | 4.469 | 3.821 | 7.120 | 0.185 | 0.740 | 0.143 |
| May | 0.107 | 1.014 | 6.917 | 84.000 | 0.469 | 4.246 | 1.183 | 1.935 | 0.126 | 0.954 | 0.231 |
| Jun | 0.126 | 1.117 | 14.271 | 88.600 | 0.440 | 4.773 | 1.536 | 0.568 | 0.156 | 1.128 | 0.164 |
| Jul | 0.200 | 1.950 | 28.800 | 202.000 | 0.750 | 9.350 | 1.500 | 0.155 | 0.118 | 1.995 | 0.026 |
| Aug | 0.125 | 1.025 | 21.448 | 76.222 | 0.636 | 4.577 | 1.068 | 1.795 | 0.177 | 1.674 | 0.320 |
| Sep | 0.576 | 1.054 | 59.803 | 75.651 | 0.621 | 4.669 | 19.355 | 1.162 | 0.201 | 1.715 | 0.262 |
| Oct | 0.139 | 0.847 | 5.687 | 92.929 | 0.222 | 4.911 | 0.927 | 1.529 | 0.199 | 1.340 | 0.339 |
| Nov | 0.349 | 1.072 | 344.590 | 154.937 | 0.907 | 4.279 | 2.177 | 6.674 | 0.643 | 1.476 | 0.557 |
| Dec | 0.532 | 0.807 | 60.666 | 109.837 | 0.742 | 4.494 | 2.638 | 1.607 | 0.716 | 2.367 | 0.359 |

**Table S4:** THM formation at 5 ºC. Error ranges are indicated as standard errors (n=3)

|  | GWTP | SWTP | FWTP | AWTP | BWTP |
| --- | --- | --- | --- | --- | --- |
| THMs (µg/L), treated water, ≤1 mg/L DOC | | | | | |
| March | 17.79±0.21 | 29.64±0.57 | 11.11±0.11 | 50.68±0.58 | 49.66±0.21 |
| June | 43.77±0.47 | 36.37±1.00 | 11.10±0.19 | 41.38±2.79 | 27.72±0.28 |
| September | 49.58±3.11 | 12.40±0.12 | 5.35±0.10 | 8.95±0.31 | 24.14±0.55 |
| December | 32.95±0.43 | 29.76±0.79 | 12.03±0.01 | 73.63±1.46 | 16.60±0.28 |
| THMs (µg/L), raw water, 1 mg/L DOC | | | | | |
| March | 26.74±0.11 | 84.19±0.73 | 26.47±0.24 | 52.75±1.19 | 37.28±0.92 |
| June | 27.12±1.28 | 52.60±2.68 | 37.66±1.88 | 41.89±0.52 | 41.71±0.76 |
| September | 33.84±0.98 | 49.81±2.69 | 32.32±1.26 | 41.79±0.67 | 35.64±0.37 |
| December | 22.45±0.43 | 47.85±0.30 | 27.09±0.69 | 39.47±0.53 | 31.03±0.29 |
| THMs (µg/L), raw water, 5 mg/L DOC | | | | | |
| March | 78.53±1.10 | n.a. | 89.04±1.31 | 175.7±2.3 | 118.3±2.2 |
| June | 95.59±2.92 | 141.4±4.6 | 104.6±0.3 | 118.1±4.2 | n.a. |
| September | 156.6±5.2 | 145.6±4.4 | 109.5±2.8 | 93.32±6.00 | n.a. |
| December | 79.46±0.52 | 161.3±3.3 | 90.64±1.33 | 158.6±0.4 | 127.4±1.1 |

**Table S5:** THMs formation at 15 ºC.

|  | GWTP | SWTP | FWTP | AWTP | BWTP |
| --- | --- | --- | --- | --- | --- |
| THMs (µg/L), treated water, ≤1 mg/L DOC | | | | | |
| March | 22.63±0.15 | 37.78±0.07 | 12.18±0.08 | 64.43±0.96 | 62.98±1.44 |
| June | 63.02±0.36 | 50.68±2.56 | 14.00±0.18 | 54.05±2.77 | 37.69±1.26 |
| September | 58.14±1.34 | 17.50±0.73 | 6.52±0.23 | 11.84±0.72 | 31.58±1.44 |
| December | 52.20±1.89 | 53.92±1.40 | 11.68±0.86 | 114.62±2.62 | 23.67±0.65 |
| THMs (µg/L), raw water, 1 mg/L DOC | | | | | |
| March | 35.76±0.07 | 132.64±4.47 | 41.31±0.54 | 74.27±1.44 | 50.18±1.20 |
| June | 38.87±1.48 | 66.00±2.14 | 53.83±1.69 | 62.87±1.62 | 57.28±1.78 |
| September | 47.32±0.83 | 61.09±1.04 | 41.60±0.03 | 55.72±1.19 | 49.05±1.30 |
| December | 29.85±0.41 | 70.48±1.79 | 38.87±0.95 | 59.18±2.08 | 43.65±1.18 |
| THMs (µg/L), raw water, 5 mg/L DOC | | | | | |
| March | 109.6±10.1 | n.a. | 140.9±1.4 | 205.7±4.5 | 167.9±3.1 |
| June | 138.9±1.7 | 137.9±3.6 | 114.8±2.7 | 137.9±5.5 | n.a. |
| September | 177.2±1.1 | 183.0±1.2 | 123.8±1.7 | 120.8±4.2 | n.a. |
| December | 128.3±4.3 | 206.6±3.9 | 145.4±1.5 | 206.5±1.8 | 200.5±3.9 |

**Table S6:** THM formation at 25 ºC.

|  | GWTP | SWTP | FWTP | AWTP | BWTP |
| --- | --- | --- | --- | --- | --- |
| THMs (µg/L), treated water, ≤1 mg/L DOC | | | | | |
| March | 24.45±0.20 | 42.54±0.97 | 14.26±0.21 | 91.25±1.25 | 77.10±2.57 |
| June | 87.37±1.77 | 69.19±3.64 | 17.03±0.19 | 64.14±3.16 | 57.86±1.14 |
| September | 98.93±1.67 | 24.73±0.63 | 7.09±0.03 | 15.29±1.10 | 40.70±0.63 |
| December | 70.94±0.72 | 67.84±0.31 | 13.73±0.09 | 139.3±2.64 | 32.34±0.55 |
| THMs (µg/L), raw water, 1 mg/L DOC | | | | | |
| March | 43.44±0.55 | 171.0±7.0 | 55.38±0.49 | 102.2±0.7 | 59.97±1.28 |
| June | 57.42±4.52 | 87.03±1.81 | 65.16±1.30 | 77.00±2.42 | 75.79±0.98 |
| September | 64.61±1.59 | 77.09±1.60 | 52.47±0.74 | 70.42±0.89 | 62.96±0.42 |
| December | 40.49±1.10 | 87.05±1.61 | 48.14±1.31 | 80.01±1.09 | 59.08±1.11 |
| THMs (µg/L), raw water, 5 mg/L DOC | | | | | |
| March | 150.5±1.8 | n.a. | 196.7±3.4 | 227.1±4.4 | 217.4±9.3 |
| June | 150.1±4.9 | 153.2±1.8 | 128.8±2.8 | 163.3±8.7 | n.a. |
| September | 188.1±5.3 | 200.8±4.2 | 137.4±2.4 | 121.5±4.1 | n.a. |
| December | 187.7±0.8 | 200.1±5.0 | 180.9±1.8 | 191.9±2.9 | 210.8±3.0 |

**Table S7:** Synthetic surface water buffer with salts content used in THMFP tests.

| Constituent | Concentration (mg/L) |
| --- | --- |
| Na^+^ | 15.2 |
| K^+^ | 2.5 |
| Ca^2+^ | 149.8 |
| Mg^2+^ | 6.9 |
| Fe^2+^ | 1.23 |
| Mn^2+^ | 0.14 |
| Al^3+^ | 0.1 |
| HCO_3_^-^ | 400 |
| SO_4_^2-^ | 3.3 |
| Cl^-^ | 44.5 |
| PO_4_^3-^ | 0.6 |
| Br^-^ | 0.09 |
| pH | 6.9-7.1 |
| TOC | 0 |

**Figure S1:** Change in trihalomethanes formation potential for different water temperatures. Error bars indicate the standard errors (n=9-20) between the measurements for waters from different case study sites and seasons, while stars indicate the significance of the z-test results for the observed reduction being different from zero (* p ≤ 0.05, ** p ≤ 0.01, *** p ≤ 0.001). BDCM = bromodichloromethane, DBCM = dibromochloromethane.


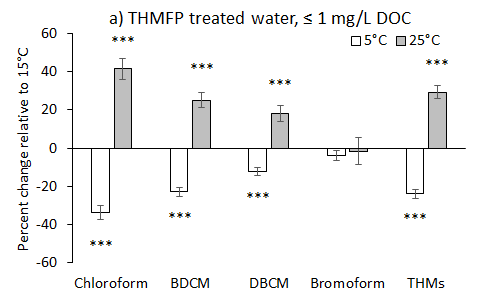


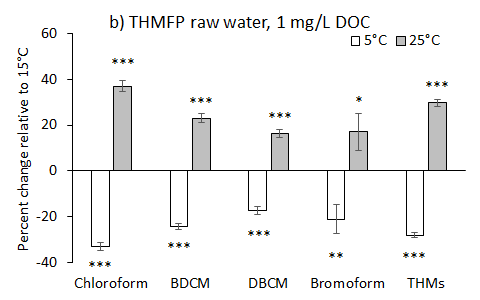


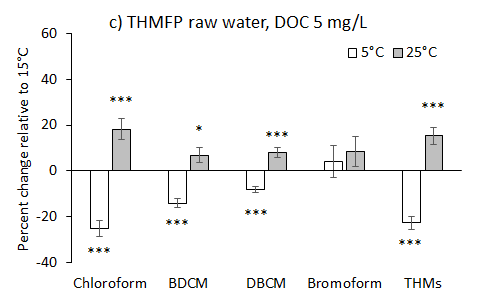


**Figure S2:** Changes in trihalomethanes (THMs) formation potential for different DOC concentrations at different temperatures. Error bars indicate the standard errors (n=12-17) between the measurements for waters from different case study sites and seasons. All the measured increases were statistically significantly different from zero (z-test, all p ≤ 0.01). BDCM = bromodichloromethane, DBCM = dibromochloromethane.


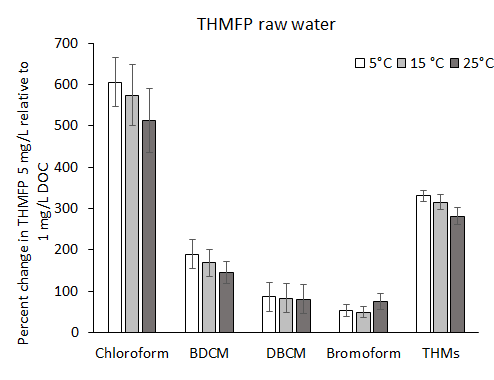

Supplement: Supplementary file 1 — Supplementary information [file 41598_2019_46238_MOESM1_ESM.docx]
